# Supplementary material for: Vitamin D Levels in Early and Middle Pregnancy and Preeclampsia, a Systematic Review and Meta-Analysis
Source: Nutrients. 2022 Feb 27;14(5):999. doi: 10.3390/nu14050999 (PMC8912474; doi:10.3390/nu14050999)
Supplement: Supplementary file 1 [file nutrients-14-00999-s001.zip › nutrients-1601538-supplementary/supplemental files/Figure S2.pdf]

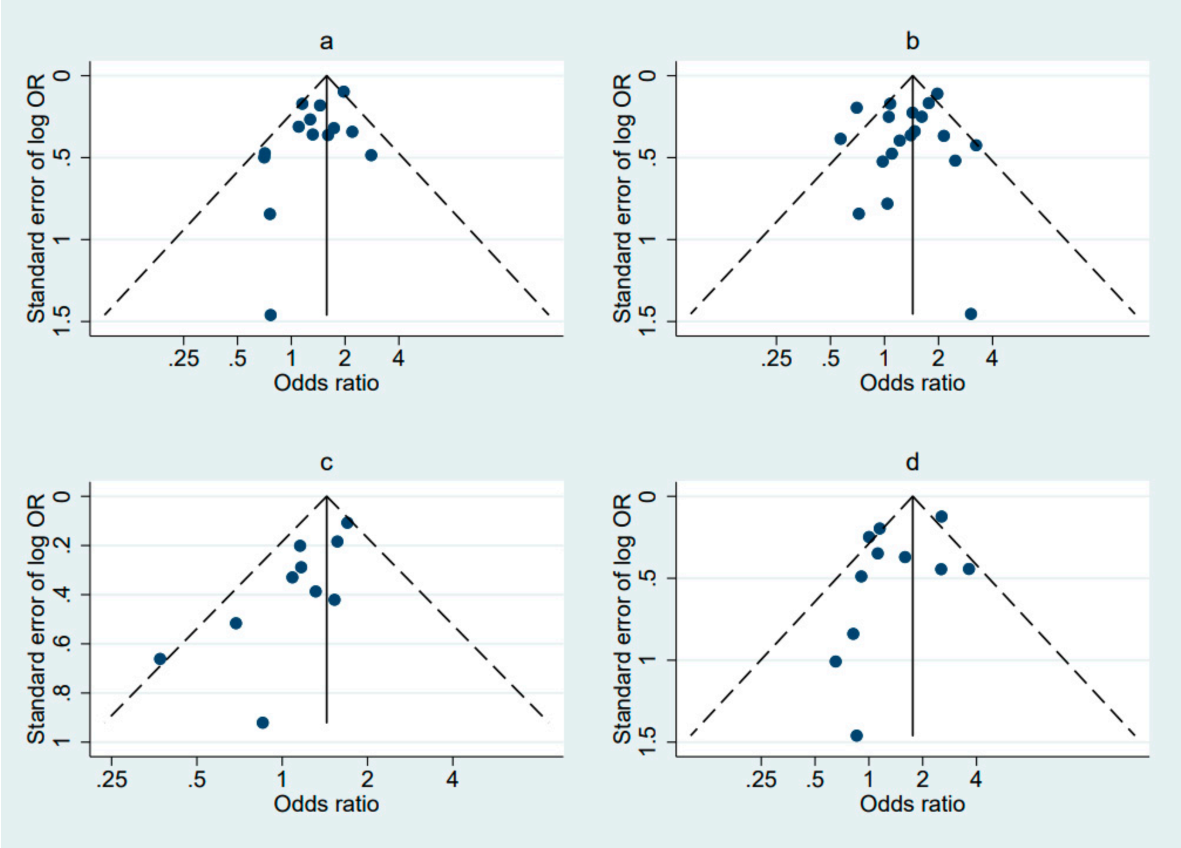

Figure S2. Funnel plot to assess publication bias. (**a**): Studies evaluating replete versus insufficient or deficient vitamin D levels; (**b**): Studies evaluating replete or insufficient versus deficient vitamin D levels; (**c**): Studies evaluating replete versus insufficient vitamin D levels; (**d**): Studies evaluating replete versus deficient vitamin D levels.
