# Supplementary material for: Vitamin D Levels in Early and Middle Pregnancy and Preeclampsia, a Systematic Review and Meta-Analysis
Source: Nutrients. 2022 Feb 27;14(5):999. doi: 10.3390/nu14050999 (PMC8912474; doi:10.3390/nu14050999)
Supplement: Supplementary file 1 [file nutrients-14-00999-s001.zip › nutrients-1601538-supplementary/supplemental files/Table S1.pdf]

**Table S1**: Search method for literature.

| **Pubmed** | |
|---|---|
| #1 | (((vitamin D[MeSH Terms]) OR (vitamin D[Title/Abstract])) OR (cholecalciferol[Title/Abstract])) OR (ergocalciferol[Title/Abstract]) |
| #2 | (pregnancy[MeSH Terms]) OR (pregnancy[Title/Abstract]) |
| #3 | ((((((((pre-eclampsia[MeSH Terms]) OR (eclampsia[MeSH Terms])) OR (Hypertension, Pregnancy-Induced[MeSH Terms])) OR (preeclampsia[Title/Abstract])) OR (hypertensive disorder of pregnancy[Title/Abstract])) OR (gestational hypertension[Title/Abstract])) OR (gestational hypertensive disorder[Title/Abstract])) OR (hypertensive disorder during pregnancy[Title/Abstract])) OR (pregnancy induced hypertension[Title/Abstract])) OR (pre-eclamptic toxaemia[Title/Abstract]) |
| A total of 441 | #1 AND #2 AND #3 |
| **Cochrane library** | |
| #1 | MeSH descriptor: [Vitamin D] explode all trees OR (vitamin D):ti,ab,kw OR (cholecalciferol):ti,ab,kw OR (ergocalciferol):ti,ab,kw |
| #2 | MeSH descriptor: [Pregnancy] explode all trees OR (pregnancy):ti,ab,kw |
| #3 | MeSH descriptor: [Pre-Eclampsia] explode all trees OR MeSH descriptor: [Eclampsia] explode all trees OR MeSH descriptor: [Hypertension, Pregnancy-Induced] explode all trees OR (preeclampsia):ti,ab,kw OR (hypertensive disorder of pregnancy):ti,ab,kw OR (gestational hypertension):ti,ab,kw OR (gestational hypertensive disorder):ti,ab,kw OR (hypertensive disorder during pregnancy):ti,ab,kw OR (pregnancy induced hypertension):ti,ab,kw OR (pre-eclamptic toxaemia):ti,ab,kw |
| A total of 134 | #1 AND #2 AND #3 |
| **Embase** | |
| #1 | 'vitamin d':ti,ab,kw OR colecalciferol:ti,ab,kw OR ergocalciferol:ti,ab,kw |
| #2 | pregnancy:ti,ab,kw |
| #3 | preeclampsia:ti,ab,kw OR 'hypertensive disorder of pregnancy':ti,ab,kw OR 'gestational hypertension':ti,ab,kw OR 'gestational hypertensive disorder':ti,ab,kw OR 'hypertensive disorder during pregnancy':ti,ab,kw OR 'pregnancy induced hypertension':ti,ab,kw OR 'pre-eclamptic toxaemia':ti,ab,kw |
| A total of 469 | #1 AND #2 AND #3 |
| **WOS** | |
| #1 | ((TS=(vitamin D)) OR TS=(cholecalciferol)) OR TS=(ergocalciferol) |
| #2 | TS=(pregnancy) |

| | |
|---|---|
| #3 | ((((((TS=(preeclampsia)) OR TS=(hypertensive disorder of pregnancy)) OR TS=(gestational hypertension)) OR TS=(gestational hypertensive disorder)) OR TS=(hypertensive disorder during pregnancy)) OR TS=(pregnancy induced |
| A total of 487 | #1 AND #2 AND #3 |
