# Supplementary material for: Vitamin D Levels in Early and Middle Pregnancy and Preeclampsia, a Systematic Review and Meta-Analysis
Source: Nutrients. 2022 Feb 27;14(5):999. doi: 10.3390/nu14050999 (PMC8912474; doi:10.3390/nu14050999)
Supplement: Supplementary file 1 [file nutrients-14-00999-s001.zip › nutrients-1601538-supplementary/supplemental files/Table S2.pdf]

Table S2: The Newcastle-Ottawa Scale (NOS) for assessing the quality of case-control studies.

Studies	Is the Case Definition Adequate	Representativeness of the Cases	Selection of Controls	Definition of Controls	Comparability of Cases and Controls		Ascertainment of Exposure	Same method of ascertainment for cases and controls	Non-Response Rate
					Age	Other factors			
Wetta, Biggio et al. (2014)	1	1	1	1	1	1	1	1	1
Baker, Haeri et al. (2010)	1	1	1	1	1	1	1	1	0
Schneuer, Roberts et al. (2014)	1	1	1	1	1	1	1	1	1
Powe, Seely et al. (2010)	1	1	1	1	0	1	1	1	0
Baca, Simhan et al. (2016)	1	1	1	1	1	1	1	1	0
Bodnar, Catov et al. (2007)	1	1	1	1	0	1	1	1	0
Benachi, Baptiste et al. (2020)	1	1	1	1	1	1	1	1	1
Achkar, Dodds et al. (2015)	1	1	1	1	1	1	1	1	0
Mirzakhani, Litonjua et al. (2016)	1	1	1	1	1	1	1	1	0
Álvarez-Fernández, Prieto et al. (2015)	1	0	1	1	0	1	1	1	0
Gidlöf, Silva et al. (2015)	1	1	1	1	0	0	1	1	1
