# Supplementary material for: Vitamin D Levels in Early and Middle Pregnancy and Preeclampsia, a Systematic Review and Meta-Analysis
Source: Nutrients. 2022 Feb 27;14(5):999. doi: 10.3390/nu14050999 (PMC8912474; doi:10.3390/nu14050999)
Supplement: Supplementary file 1 [file nutrients-14-00999-s001.zip › nutrients-1601538-supplementary/supplemental files/Table S3.pdf]

Table S3: The Newcastle-Ottawa Scale (NOS) for assessing the quality of cohort studies.

Studies	Representativeness of the exposed cohort	Selection of the non exposed cohort	Ascertainment of exposure	Demonstration that outcome of interest was not present at start of study	Comparability of cohorts		Assessment of outcome	Was follow-up long enough for outcomes to occur	Adequacy of follow up of cohorts
					Age	Other factors			
Zhou, Su et al. (2014)	1	1	1	1	1	1	1	1	1
Hemmingway, Kenny et al. (2018)	1	1	1	1	1	1	1	1	1
Wei, Audibert et al. (2012)	1	1	1	1	1	1	1	1	1
Flood-Nichols, Tinnemore et al. (2015)	1	1	1	0	0	1	1	1	1
Shand, Nassar et al. (2010)	0	1	1	1	1	1	1	1	1
Van Weert, van den Berg et al. (2016)	1	1	1	1	1	1	1	1	1
Magnus, Miliku et al. (2018)	1	1	1	1	1	1	1	1	1
Scholl, Chen et al. (2013)	0	1	1	1	1	1	1	1	1
