# Supplementary material for: Vitamin D Levels in Early and Middle Pregnancy and Preeclampsia, a Systematic Review and Meta-Analysis
Source: Nutrients. 2022 Feb 27;14(5):999. doi: 10.3390/nu14050999 (PMC8912474; doi:10.3390/nu14050999)
Supplement: Supplementary file 1 [file nutrients-14-00999-s001.zip › nutrients-1601538-supplementary/supplemental files/Table S4.pdf]

Table S4. AHQR for assessing the quality of cross-sectional studies.

Studies	Define the source of information	List inclusion and exclusion criteria	Indicate time period used for identifying patients	Indicate whether or not subjects were consecutive	Indicate if evaluators were masked to other aspects of the status of the participants	Describe any assessments for quality assurance purposes	Explain any patient exclusions from analysis	Describe how confounding was assessed and/or controlled	If applicable, explain how missing data were handled in the analysis	Summarize patient response rates and completeness of data collection	Clarify what follow-up was expected and the percentage of patients for which incomplete data or follow-up was obtained	Total score
Fernández-Alonso, Dionis-Sánchez et al. (2012)	1	1	1	1	1	1	0	0	0	0	1	7
Christoph, Challande et al. (2020)	1	1	1	1	1	1	0	0	0	0	1	7
Al-Shaikh, Ibrahim et al. (2016)	1	1	1	1	1	1	0	0	0	1	1	8
