# Supplementary material for: Vitamin D Levels in Early and Middle Pregnancy and Preeclampsia, a Systematic Review and Meta-Analysis
Source: Nutrients. 2022 Feb 27;14(5):999. doi: 10.3390/nu14050999 (PMC8912474; doi:10.3390/nu14050999)
Supplement: Supplementary file 1 [file nutrients-14-00999-s001.zip › nutrients-1601538-supplementary/supplemental files/Table S5.pdf]

Table S5. Vitamin D cutoff values and the calculated rate of preeclampsia in the included studies.

Author (year)	Vitamin D cutoff	Calculated rate	Confounding factors identified in each studies
Wetta, Biggio et al. (2014)	≥30<br>15-30<br><15 ng/ml<br>(<30 ng/mL)	30.3% (33/109)<br>33.6% (38/113)<br>40.9% (18/44)<br>34.8% (56/157)	Age, race/ethnicity, parity, history of a prior preterm birth, maternal weight at first prenatal visit, smoking status, gestational age at which the sample was drawn and time of year specimen obtained
Baker, Haeri et al. (2010)	≥75<br>50-75<br><50 nmol/liter	13.8% (22/160)<br>19.6% (10/51)<br>36.7% (11/30)	Season of blood draw, maternal age, multiparity, body mass index, and gestational age at serum collection
Zhou, Su et al. (2014)	≥30<br>21-29<br><20 ng/mL	3.51% (13/370)<br>3.81% (36/946)<br>3.92% (25/637)	Maternal age, systolic/diastolic pressure, prepregnancy body mass index and serum calcium
Schneuer, Roberts et al. (2014)	>75<br>37.5-75<br><37.5 nmol/L	3.7% (37/999)<br>5.7% (186/3275)<br>3.7% (31/835)	Maternal age, parity, smoking during pregnancy, maternal weight, previously diagnosed hypertension, previously diagnosed diabetes, season at sampling, country of birth, or socioeconomic disadvantage
Powe, Seely et al. (2010)	>15<br><15 ng/mL	12.5% (5/40)<br>26.2% (34/130)	Body mass index, nonwhite race, and summer blood collection
Baca, Simhan et al. (2016)	>75<br>50-75<br><50 nmol/L	20.5% (209/1018)<br>30.5% (261/856)<br>39.7% (180/453)	Year of delivery, laboratory batch number, gestational age and season at blood drawn, race/ethnicity, maternal age, smoking status, prepregnancy BMI, insurance, education, marital status, and parity
Bodnar, Catov et al. (2007)	>75<br>37.5-75<br><37.5 nmol/liter	14.0% (12/86)<br>17.6% (23/131)<br>29.2% (14/48)	Race/ethnicity, season, sample, gestational age, prepregnancy BMI, and education.
Benachi, Baptiste et al. (2020)	<30<br>≥30 ng/mL	22.6% (69/305)<br>14.4% (14/97)	Parity, maternal age, pre-pregnancy BMI, season of conception, skin color
Achkar, Dodds et al. (2015)	≥50<br>30-50<br>≤30 nmol/L<br>(<50 nmol/L)	6.0% (65/1092)<br>9.2% (72/779)<br>13.6% (24/177)<br>10.0% (96/956)	Pre-pregnancy body mass index, parity, maternal age, smoking, season of blood collection, year of blood collection, gestational age at blood collection, and study site
Fernández-Alonso, Dionis-Sánchez et al. (2012)	≥30<br>21-29<br>≤20 ng/mL	1.8% (2/109)<br>1.6% (3/191)<br>1.2% (2/166)	None
Christoph, Challande et al. (2020)	≥50<br><50 nmol/L	1.6% (6/370)<br>1.8% (18/1012)	None
Al-Shaikh, Ibrahim et al. (2016)	≥30<br>21-29<br>≤20 ng/mL	0% (0/38)<br>0% (0/98)<br>1.0% (9/864)	None
Hemmingway, Kenny et al. (2018)	≥75<br><75 nmol/L	2.3% (10/441)<br>3.0% (39/1313)	BMI, maternal age, university education, and supplementation
Mirzakhani, Litonjua et al. (2016)	<30<br>≥30 ng/mL	34.4% (41/119)<br>15.8% (6/38)	Gravidity, age, race, BMI, study group designation, education level, income, and study center
Wei, Audibert et al. (2012)	≥50<br><50 nmol/L	4.0% (17/425)<br>5.5% (15/272)	Maternal age, smoking, prepregnancy body mass index and season of blood draw
Flood-Nichols, Tinnemore et al. (2015)	>30<br>21-29<br><20 ng/mL	10.0% (7/70)<br>7.1% (10/141)<br>8.3% (2/24)	BMI, season, ethnicity, and tobacco use
Shand, Nassar et al.	>75	15.9% (7/44)	Maternal age, ethnicity, parity, BMI, season, multivitamin use and

(2010)	50-75<br><50 nmol/liter	6.6% (4/61)<br>14.7% (17/116)	smoking status
Van Weert, van den Berg et al. (2016)	>50<br>30-50<br><30 nmol/liter	3.4% (43/1263)<br>4.3% (19/442)<br>5.4% (20/369)	Age, ethnicity, pre-pregnancy BMI, smoking and years of education after primary school.
Magnus, Miliku et al. (2018)	>75<br>50-75<br><50 nmol/liter	4.4% (54/1232)<br>5.0% (50/993)<br>5.0% (55/1098)	Age, parity, pre-pregnancy BMI, education, smoking, calcium level/calcium intake and gestational week of blood sampling
Scholl, Chen et al. (2013)	⩾20.0<br>< 20.0 ng/mL	5.1% (38/750)<br>7.9% (31/391)	Age, parity, ethnicity, BMI, smoking, and gestation at entry
Álvarez-Fern á ndez, Prieto et al. (2015)	⩾50<br><50 nmol/L	28.6% (6/21)<br>28.1% (34/121)	Uric acid and aspartate aminotransferase concentrations, multiple gestation and intrauterine growth restriction
Gidlöf, Silva et al. (2015)	⩾50<br><50 nmol/L	28.4% (23/81)<br>18.4% (14/76)	None
